# Parental body mass index and blood pressure are associated with higher body mass index and blood pressure in their adult offspring: a cross‐sectional study in a resource‐limited setting in northern Peru

**DOI:** 10.1111/tmi.13052

**Published:** 2018-04-01

**Authors:** Rodrigo M. Carrillo‐Larco, Antonio Bernabé‐Ortiz, Víctor G. Sal y Rosas, Katherine A. Sacksteder, Francisco Diez‐Canseco, María K. Cárdenas, Robert H. Gilman, J. Jaime Miranda

**Affiliations:** ^1^ CRONICAS Center of Excellence in Chronic Diseases Universidad Peruana Cayetano Heredia Lima Perú; ^2^ Department of Epidemiology and Biostatistics School of Public Health Imperial College London London UK; ^3^ Faculty of Epidemiology and Population Health London School of Hygiene and Tropical Medicine London UK; ^4^ Department of Science Pontificia Universidad Católica del Perú Lima Perú; ^5^ Department of International Health Bloomberg School of Public Health Johns Hopkins University Baltimore USA; ^6^ Área de Investigación y Desarrollo Asociación Benéfica PRISMA Lima Perú; ^7^ Department of Medicine School of Medicine Universidad Peruana Cayetano Heredia Lima Perú

**Keywords:** body mass index, overweight, obesity, blood pressure, hypertension, family health, indice de masse corporelle, surpoids, obésité, tension artérielle, hypertension, santé familiale

## Abstract

**Objectives:**

High body mass index (BMI) and blood pressure (BP) are major contributors to the high burden of non‐communicable diseases in adulthood. Individual high‐risk and population approaches for prevention require newer strategies to target these risk factors and focusing on the family to introduce prevention initiatives appears as a promising scenario. Characterisation of the relationship between BMI and BP among the adult members of a given family merits evaluation. We conducted a secondary analysis of an implementation study in Tumbes, Peru, benefiting from data derived from families with at least one adult offspring.

**Methods:**

The exposures of interest were the BMI, systolic BP (SBP) and diastolic BP (DBP) of the mother and father. The outcomes were the BMI, SBP and DBP of the offspring. Mixed‐effects linear regression models were conducted.

**Results:**

The mean age of the offspring, mothers and fathers was 29 (SD: 9.5), 54 (SD: 11.8) and 59 (SD: 11.6) years, respectively. Father's BMI was associated with a quarter‐point increase in offspring BMI, regardless of the sex of the offspring. Mother's BMI had a similar effect on the BMI of her sons, but had no significant effect on her daughters’. Mother's SBP was associated with almost one‐tenth of mmHg increase in the SBP of the adult offspring. There was no evidence of an association for DBP.

**Conclusions:**

In families with adult members, the higher the parents’ BMI and SBP, the higher their adult offspring's levels will be.

## Abstract (fr)

**Objectifs:**

L'indice de masse corporelle (IMC) et la tension artérielle (TA) élevés sont les principaux facteurs contribuant à la charge élevée des maladies non transmissibles à l’âge adulte. Les approches visant les individus à haut risque et les populations pour la prévention nécessitent de nouvelles stratégies pour cibler ces facteurs de risque et, se concentrer sur la famille pour introduire des initiatives de prévention apparaît comme un scénario prometteur. La caractérisation de la relation entre l’IMC et la TA parmi les membres adultes d'une famille donnée mérite d’être évaluée. Nous avons effectué une analyse secondaire d'une étude d'implémentation à Tumbes, au Pérou, bénéficiant de données provenant de familles ayant au moins une progéniture adulte.

**Méthodes:**

Les expositions d'intérêt étaient l’IMC, la TA systolique (TAS) et diastolique (TAD) de la mère et du père. Les résultats ont été l’IMC, TAS et TAD de la progéniture. Des modèles de régression linéaire à effets mixtes ont été effectuées.

**Résultats:**

L’âge moyen de la progéniture, des mères et des pères était respectivement de 29 ans (SD: 9,5), 54 ans (SD: 11,8) et 59 ans (SD: 11,6). L’IMC du père était associé à une augmentation de l’IMC de la progéniture d'un quart de point, quel que soit le sexe de la progéniture. L’IMC de la mère avait un effet similaire sur l’IMC de ses fils, mais pas d'effet significatif sur ses filles. La TAS de la mère était associée à une augmentation d'environ un dixième de mmHg de la TAS de la progéniture adulte. Il n'y avait aucune preuve d'association pour la TAD.

**Conclusions:**

Dans les familles comptant des membres adultes, plus l’IMC et la TAS des parents sont élevés, plus ils seront élevés chez leur descendance adulte.

## Introduction

Over the last decades, mortality due to non‐communicable diseases (NCDs) has increased globally, with cardiovascular diseases at the top of the list 1. Worldwide trends show that body mass index (BMI) and obesity rates 2, 3, as well as high blood pressure (BP) alongside with diastolic and systolic blood pressure (DBP/SBP) 4, have increased between 1975 and 2015. In 2015, high SBP and BMI were among the leading contributors to disability‐adjusted life‐years 5. The impact of high BMI and high BP on NCDs is not trivial, and it challenges economies and societies.

The epidemiology of these risk factors – BMI and high BP – is not encouraging in developing countries, where there are more deaths attributable to NCDs than in developed nations 6. Mean BMI in Peru, a middle‐income country, in 2014 was approximately 26.5 kg/m^2^ 3, a population average estimate in the range of overweight. A longitudinal study conducted between 2007–2008 and 2012–2013 among Peruvian rural‐to‐urban migrants, rural and urban subjects showed that the obesity incidence in these three groups was 2.3, 0.4 and 2.6 per 100 person‐years, respectively 7. This suggests that obesity rates are to increase particularly in urbanised sites and those undergoing rapid urbanisation. In contrast, starting from a baseline hypertension prevalence of 19.7%, a population‐based Peruvian study reported a hypertension incidence of 7.12 new cases per 100 person‐years, with different risk estimates according to degree of urbanisation; semi‐urban sites exhibited the highest risk 8. These BMI and BP estimates at the population level reveal a complex scenario for NCDs, yet evaluation of these risk factors at the family level, particularly among its adult members, could signal additional windows of opportunity for prevention initiatives. Interventions at the family level have shown promising results in targeting and improving negative lifestyles related to sedentarism 9, 10, diet and weight 11, 12 of the household's children; however, family health of adult subjects has been understudied. Whilst individual‐level lifestyle recommendations to improve cardiovascular health are important 13, new interventions considering a whole‐family approach can be proposed to reduce weight. Moreover, family health research contributes to understanding the developmental origins of health and disease hypothesis, which still needs to be acknowledged in clinical practice 14.

Although the intrafamily relationship of health risk factors could be understood on genetic studies, in the era of evidence‐based medicine, these are not enough to conclude in favour of (or against) interventions. Observational epidemiological studies, although limited in terms of causality assessment, do provide a first step to describe whether there is a health issue in the study population, worthwhile of intervention. Consequently, we aimed to characterise the relationship between parental BMI, SBP and DBP and that of their adult offspring's.

## Methods

### Study design and setting

This was a cross‐sectional analysis using data of the baseline assessment of a population‐based implementation study in Peru; further details about the implementation study can be found elsewhere 15. The study was conducted in Tumbes, a semi‐urban setting at sea level in northern Peru. Figures for 2016 revealed that there were 240 590 people in Tumbes, with a life expectancy of 74.7 years; 18.9% reported not having any health insurance, and 9.8–12.6% of the population was under the poverty line 16.

### Study population

The population included in this study is the same one enrolled at baseline in the implementation study;^15^ there were no differences between our study population and the one of the trial study. Subjects were enrolled from six randomly selected rural villages. All members of the household aged ≥18 years were included in the implementation study. The exclusion criteria comprised having any mental illness that would prevent giving informed consent, self‐reported chronic kidney disease and self‐reported heart disease 15.

Because of the inclusion criteria in the implementation study, only adult subjects were analysed herein. For this study, we included subjects with complete information on BMI, DBP and SBP. Only one family was included per household (first family in the registry) with at least father or mother, regardless of the number of adult offspring, although each family had to have at least one. Thus, in the analysis, there could have been single‐parent families, nuclear families or families with several children. The number of participants included in the study is available in Figures S1 and S2, for DBP/SBP and BMI, respectively; also, these figures show the process conducted to identify family relationships/members; first, we cleaned data looking for fathers, mothers and children within the same house; of these, we only kept those with complete data on the outcomes of interest; lastly, we analysed families with at least father or mother and one offspring.

### Variables

The clinical evaluation was conducted by trained fieldworkers following standardised procedures 15. BP was assessed three times in a resting position after a five‐minute resting period, with at least one minute between measurements. For this study, the mean of the last two readings was used. Weight and height were measured with the participants wearing light clothes and no shoes. All other variables were elicited in a paper‐based interview using validated questions, such as those from the WHO STEPS approach 15, 17.

#### Outcome variables

The outcomes were BMI (kg/m^2^), DBP (mmHg) and SBP (mmHg) of the offspring in each family, assessed as continuous variables.

#### Exposure variables

The exposures were BMI (kg/m^2^), DBP (mmHg) and SBP (mmHg) of mothers and fathers at each family. These variables were treated as continuous variables.

#### Other variables

Other variables included in the study were as follows: village in which the participant lived (categorical variable); sex; age (continuous variable); education level (none/primary, secondary and higher education); physical activity assessed with the short version of the International Physical Activity Questionnaire (IPAQ) and classified as low, moderate or high;^18^ heavy drinker was defined as having a hangover or ≥6 drinks at one occasion at least once per month (Yes/No); current smoker (Yes/No); if the subject added salt to the food when eating (never or at least some times); and father's wealth index, which is the same for the family, and it is a composite numeric index based on facilities and goods owned by the household 19. Of these, the following were included in the adjusted regression models: offspring's sex, age, physical activity, educational level, father's wealth index and village in which the participant lives; height (offspring's, father's and mother's) was included when BP was the outcome of interest. Other variables (e.g. parental physical activity) were not adjusted for because of strong correlation between parents.

### Statistical analysis

Lineal mixed regression models, with two hierarchical levels (individual and family) and an unstructured covariance matrix, were used to assess whether the BMI, DBP and SBP of the parents were associated with the BMI, DBP and SBP of the offspring. Factors that confounded or modified this relationship were included in a multivariate analysis such as age, sex, education level, height (offspring, father and mother), socioeconomic status (of the family) and study village. Analyses were conducted with STATA v13.0 (StataCorp, College Station, TX, USA)

In a pre‐specified analysis, sex of the offspring was included as an effect modifier of the relationship between BMI/DBP/SBP of the offspring and the BMI/DBP/SBP of the father and the mother, separately. To account for the situation where offspring currently have only a mother (94, 21.3%) or only a father (30, 6.8%), two indicator variables of whether the family has current a father or a mother were included in the model. Even though families without a father cannot be used to estimate the effect of BMI/SPB/DBP of the father on the offspring, they can still be used to estimate the effect of the other covariates.

Results are presented as the mean difference, at the population level, associated with a one unit increment in the covariate, conditionally on having a father or a mother. 95% confidence intervals were presented for all estimations. Details about the fitted model are available as Appendix S1.

### Ethics

The implementation study was approved by the Institutional Review Boards at Universidad Peruana Cayetano Heredia (Lima, Peru) and Johns Hopkins University (Baltimore, USA). Signed informed consent was obtained from all study participants 15.

## Results

### Characteristics of the study population

There were 955 subjects who met our inclusion criteria; 438 offspring (253 males and 185 females) were available for analysis along with 237 fathers and 280 mothers. The median size of a family was three members [IQR = 3–4, Range = 2–6]. The mean number of offspring per family was 1 [IQR = 1–2, range = 1–4]. The mean age of the offspring, mothers and fathers was 29 (SD: 9.5), 54 (SD: 11.8) and 59 (SD: 11.6) years, respectively (*P* < 0.001, Table 1).

**Table 1:** Sociodemographic and clinical characteristics of the studied families

	Overall	Father	Mother	Male offspring	Female offspring
Sex					
Female	465 (48.7%)	0 (0.0%)	280 (100.0%)		185 (100.0%)
Male	490 (51.3%)	237 (100.0%)	0 (0.0%)	253 (100.0%)	
Age					
Mean (SD)	44 (17.7)	59 (11.6)	54 (11.8)	29 (9.5)	29 (9.5)
Median (IQR)	44 (27–57)	58 (51–67)	53 (46–61)	26.5 (22–32)	27 (22–34)
Education					
None/primary	370 (38.7%)	150 (63.3%)	183 (65.4%)	23 (9.1%)	14 (7.6%)
Secondary	393 (41.2%)	70 (29.5%)	80 (28.6%)	162 (64.0%)	81 (43.8%)
Higher	192 (20.1)	17 (7.2%)	17 (6.1%)	68 (26.9%)	90 (48.7%)
Wealth index					
Bottom	317 (33.5%)	–	–	–	
Middle	317 (33.5%)	–	–	–	
Top	312 (33.0%)	–	–	–	
Physical activity					
Low	616 (64.5%)	105 (44.3%)	238 (85.0%)	133 (52.6%)	140 (75.7%)
Moderate	254 (26.6%)	93 (39.2%)	38 (13.6%)	84 (33.2%)	39 (21.1%)
High	85 (8.9%)	39 (16.5%)	4 (1.4%)	36 (14.2%)	6 (3.2%)
Heavy drinker					
No	881 (92.3%)	217 (91.6%)	278 (99.3%)	202 (79.8%)	184 (99.5%)
Yes	74 (7.8%)	20 (8.4%)	2 (0.7%)	51 (20.2%)	1 (0.5%)
Current smoker					
No	852 (89.5%)	173 (73.6%)	279 (99.6%)	216 (85.7%)	184 (99.5%)
Yes	100 (10.5%)	62 (26.4%)	1 (0.4)	36 (14.3%)	1 (0.5%)
Add salt					
Never	878 (92.0%)	218 (92.0%)	262 (93.6%)	229 (90.5%)	169 (91.9%)
At least some times	76 (8.0%)	19 (8.0%)	18 (6.4%)	24 (9.5%)	15 (8.1%)
BMI					
Mean (SD)	26.7 (4.8)	26.6 (4.4)	29.1 (4.8)	25.1 (4.1)	25.6 (4.7)
Median (IQR)	26.5 (23.3–29.8)	26.1 (23.4–29.6)	29.3 (25.9–32.4)	24.6 (21.8–27.9)	25.3 (22.0–28.4)
Height					
Mean (SD)	159 (8.8)	164.9 (6.3)	152.2 (5.9)	166.8 (6.3)	155.3 (6.2)
Median (IQR)	159.5 (153.2–166.4)	165.2 (160.7–169.4)	152.2 (148.3–156.0)	167.0 (163.0–171.2)	155.2 (151.8–158.7)
DBP					
Mean (SD)	72.7 (10.3)	75.4 (10.7)	74.0 (10.8)	71.7 (9.6)	68.6 (8.4)
Median (IQR)	71.5 (65.5–78.0)	74.0 (67.5–81.5)	72.0 (66.5–80.5)	71.0 (65.5–76.5)	68.0 (63.5–73.5)
SBP					
Mean (SD)	113.8 (17.0)	120.7 (17.2)	116.0 (19.5)	114.2 (11.9)	101.4 (10.9)
Median (IQR)	111.5 (103.0–122)	119.5 (109.5–129.0)	112.5 (103.0–125.5)	112.5 (107.0–119.5)	99.5 (94.5–106.0)

BMI, body mass index; DBP, diastolic blood pressure; SBP, systolic blood pressure. Wealth index is the same at the household level.

### Family aggregation: BMI

In the adjusted model, father's and mother's BMI was associated with higher offspring's BMI (Table 2). For each additional father's BMI unit, the offspring's BMI increased on average 0.25 [95% CI 0.13; 0.37] kg/m^2^; there was no interaction between the father's BMI and the offspring sex. The BMI of the mother was associated with an average increase of 0.20 [95% CI 0.10; 0.31] in the BMI of the male offspring, whilst this figure for the female offspring was not significant. This suggests that the mother's BMI has a stronger influence on the BMI of her sons than on the BMI of her daughters.

**Table 2:** Association between BMI/SBP/DBP of parents and BMI/SBP/DBP of their offspring using a multilevel mixed‐effects linear regression model (*n* = 442; cluster = 308)

	Crude estimates		Adjusted estimates	
	β	95% CI	β	95% CI
Model 1: BMI as outcome				
Father–Female Offspring	**0.19**	**0.07;0.32**	**0.25**	**0.13;0.37**
Father–Male Offspring	**0.20**	**0.08;0.32**	**0.26**	**0.14;0.38**
Mother–Female Offspring	0.10	−0.02;0.21	0.11	−0.00;0.22
Mother–Male Offspring	**0.20**	**0.10;0.30**	**0.20**	**0.10;0.31**
Model 2: SBP as outcome				
Father–Female Offspring	0.06	−0.02;0.14	0.05	−0.03;0.12
Father–Male Offspring	0.05	−0.03;0.12	0.03	−0.04;0.11
Mother–Female Offspring	**0.11**	**0.04;0.18**	**0.09**	**0.03;0.17**
Mother–Male Offspring	**0.13**	**0.07;0.19**	**0.11**	**0.05;0.18**
Model 3: DBP as outcome				
Father–Female Offspring	**0.10**	**0.01;0.20**	0.10	−0.00;0.19
Father–Male Offspring	0.09	−0.01;0.19	0.09	−0.01;0.18
Mother–Female Offspring	0.06	−0.04;0.16	0.07	−0.03;0.17
Mother–Male Offspring	0.08	−0.02;0.17	0.08	−0.02;0.17

Adjusted by village, age, educational level, physical activity of the offspring and wealth index of the family. For the blood pressure models, height of offspring, mother and father was also included. Each line represents the mean effect in the outcome variable in the offspring that the same variable of the father or mother has. For example, in model 1, Father–female offspring measured the mean change in the BMI of the female offspring given a one unit change in the BMI of the father, adjusting for other covariates. Bold values are statistical significant at a threshold of *P*<0.05.

### Family aggregation: Blood Pressure

In the adjusted model, an increase in mother's SBP was associated with a mean increment of 0.11 [95% CI 0.05; 0.18] on SBP of the female offspring. Neither father's DBP/SBP nor mother's DBP/SBP significantly interacted with the offspring sex, suggesting there was no same‐sex or cross‐sex resemblance between parents and offspring regarding BP.

## Discussion

### Main findings

In semi‐urban resource‐limited villages in northern Peru, as the parent's BMI increased, so did the male and female offspring's BMI; the mother's BMI had a bigger effect on her son's BMI than on her daughter's. As the mother's SBP rose, so did the offspring's SBP. These results suggest a resemblance between adult offspring and parents in BMI and SBP. Although the study population was healthy subjects and the magnitude of the association estimates herein described was small, the overall resemblance should not be overlooked because even within a normal range of BMI and BP, there is a trend towards higher values of these risk factors.

### Public health implications

Although restricted by the study design and other limitations, the results may call to implement prevention strategies at the family level to avoid high BMI and BP ‘contagion’ among members, particularly regarding BMI, for which the association estimates were stronger. The scope of our paper is beyond advocating for a particular intervention, yet there are a few examples that could be used to improve cardiovascular risk factors at the family level. Interventions could target the environment or subjects in the household, addressing portion size and table/dishware features could foster less food consumption 20, presumably having a weight reduction effect 21, 22. Although the proposed interventions would target all family members, others can be aimed at some members alone. A recent trial showed that a family‐based intervention was non‐inferior to a parent‐based intervention to reduce weight in the children 12, suggesting that not all family members ought to be actively involved in the intervention.

### Comparison with previous results: BMI

Our results showed that higher BMI of both parents was associated with higher BMI of the offspring. Other studies have shown similar results 23, 24, 25. A study in China with 23–24‐year‐old offspring reported that, as father's and mother's BMI increased, so did the offspring's 23. An Australian study reached a similar conclusion, but also assessing other obesity indicators (e.g. waist circumference) 25. Comparable figures were retrieved in the United Kingdom too 24. Also, some studies have suggested that the BMI resemblance may follow a same‐sex pattern: father–son and mother–daughter 24, 25, 26. However, other authors have reported a BMI resemblance between father–daughter 27 and mother–son 28. Our results suggest that the mother's BMI greatly influences that one of her sons. If the mother's BMI has a stronger correlation with the son's BMI, it could be because of a biased view that males must be robust as a synonym of good health 29, 30. These beliefs should be taken into consideration when addressing weight loss in the study population, or others with Latino background 31.

### Comparison with previous results: blood pressure

Our results showed that there was a correlation between mother's SBP and that of the offspring; nevertheless, results were not conclusive regarding father's SBP or either parent DBP. Previous reports have yielded more conclusive results 23, 32. A study in Korea with adolescents revealed a positive BP correlation between parents and children; of note, regarding the dyads mother–son and mother–daughter, the correlation was significant for DBP only 32. With regard to adults, a study with Chinese Han population revealed that higher DBP or SBP of the father and mother was associated with higher SBP in the offspring 23. A possible explanation for our discrepant results could be lack of statistical power because some confidence intervals were close to significance. Another possible explanation could come from the profile of salt added at the table, which was not different among family members. Thus, approximately everybody in the family has the same salt intake. Nevertheless, one limitation of this proposed interpretation is that food consumed outside the house was not analysed. Future studies need to address all possible salt sources in the daily diet.

### Interpretation of results

The associations between parents and offspring regarding the assessed risk factors could be due to genetic reasons 33, 34, or to shared environmental features. Johnson et al 24. suggested that the different size effects between mother/father and offspring regarding BMI could be explained by genetic factors, but most importantly by environmental ones, because same‐sex transmission of genes is not the commonest Mendelian trait 23, 35. Then, the same‐sex resemblance could be explained by factors such as physical activity or diet profiles. Also, interactions between family members, family functioning and parenting styles 36, as well as social networks between grownup siblings^37^ could explain the results because of the effects one family member has on the others. It is worth mentioning that the offspring assessed in this study still live with their parents, and most likely they have all lived together since ever. Because health‐related behaviours or lifestyles are learnt throughout their lifetime, prevention strategies should be applied early in life. It has been reported a stronger correlation between adiposity indexes between parents and adolescent offspring, than between parents and children 38. This could suggest that adolescents have already acquired unhealthy lifestyles, which supports starting prevention strategies at early ages. In addition to this potential resemblance of unhealthy health behaviours, evidence in developmental origins of health and diseases further supports looking for and managing the higher risk of non‐communicable diseases early in life 14.

The literature suggests that maternal traits affect equally offspring traits, and with a larger effect than paternal traits 39. Our results partially challenge this statement because, for example, the effect of mother's BMI seems to be larger in sons than daughters. We hypothesise this is because parenting style as well as cultural background, and the perception that males need to be robust as a synonym of good health, most likely occur in Hispanic backgrounds 29. Therefore, variability in the effect of father's and mother's traits could depend on (biased) perceptions of weight and health standards. Overall, our results support the findings of previous studies and recently summarised by Devakumar *et al*. 39, suggesting that parental traits influence sons and daughter differently.

### Strengths and limitations

This study had some strengths. First, we studied families from the general population in a resource‐limited setting in Latin America. Second, because BP is the main outcome of the implementation study from which the study sample was drawn 15, it was assessed thoroughly, and so was BMI. Nonetheless, limitations must be highlighted too. First, the study sample was not enrolled for this study in particular; thus, the results could be underpowered and may not represent the targeted population which could bias the results. However, we still present strong associations suggesting our results are robust and probably alert there is a much greater effect. Likewise, because the original trial did not include people who reported kidney or heart diseases, these individuals were not included in this study. Generalisability of our results to families with these patients may be limited. Second, there could be residual confounding; also, due to data availability, we could not include other potential confounders such as diet profile (e.g. fruits/vegetable consumption). Nevertheless, because we included members of the same family living together, they probably shared a similar diet so adjusting for this variable would not have had a strong effect on the results. In spite of these limitations and the cross‐sectional design of the analysis, the results showed a strong pattern that deserves to be studied longitudinally to verify whether parental behaviours or characteristics influence the offspring enough to develop high BMI or BP. Third, we did not have data on non‐paternity and we did not conduct sensibility analysis to address this possibility. Devakumar *et al*., in a sensibility analysis, revealed that with increasing levels of non‐paternity, the point estimates of the association between parent and offspring BMI slightly decreased for mothers and scarcely increased for fathers 39. This signals the potential health relevance of further studying non‐communicable diseases at the family level and with different family structures, that is, including non‐paternity profiles. Fourth, unlike other studies which focused on complete families (i.e. father, mother and child), our regression models accounted for the absence of either the father or the mother. Rather than a limitation, this procedure improved data efficiency, maximising our sample size without the need to undertake imputation methods. When comparing our results with those of a complete‐case analysis (i.e. including complete families), the estimates were very similar.

## Conclusions

Higher parent's BP (systolic) and BMI are associated with higher BP (systolic) and BMI of the adult offspring living in the same family. The family level seems to be a feasible scenario to display prevention strategies to reduce aggregation of high BMI and BP among family members.

## Supporting information


**Figure S1.** Number of subjects included in the study, outcome: blood pressure.
**Figure S2.** Number of subjects included in the study, outcome: BMI.

**Appendix S1.** Fitted Regression Model.
