# Supplementary material for: Parental body mass index and blood pressure are associated with higher body mass index and blood pressure in their adult offspring: a cross‐sectional study in a resource‐limited setting in northern Peru
Source: Trop Med Int Health. 2018 Apr 1;23(5):533–40. doi: 10.1111/tmi.13052 (PMC5932220; doi:10.1111/tmi.13052)
Supplement: Supplementary file 1 — Figure S1. Number of subjects included in the study, outcome: blood pressure. Figure S2. Number of subjects included in the study, outcome: BMI. [file TMI-23-533-s001.docx]

## Supplementary Figure 1: Number of subjects included in the study, outcome: blood pressure.

Subjects at baseline

N=2,376

N=2,241

Keep only fathers, mothers and offspring

N=2,222

N=2,165

Keep only one family per household

Keep subjects with complete data on BMI

N=2,154

Exclude incongruences (≥1 father or mother in the family)

Both parents

N=318

Only Fathers

N=30

Only Mothers

N=94

Families with at least father or mother and one offspring

Offspring

N=442

## Supplementary Figure 2: Number of subjects included in the study, outcome: BMI.

Subjects at baseline

N=2,376

N=2,241

Keep only fathers, mothers and offspring

N=2,222

N=2,165

Keep only one family per household

Keep subjects with complete data on BMI

N=2,154

Exclude incongruences (≥1 father or mother in the family)

Both parents

N=311

Only Fathers

N=32

Only Mothers

N=95

Families with at least father or mother and one offspring

Offspring

N=438
