# Supplementary material for: Parental body mass index and blood pressure are associated with higher body mass index and blood pressure in their adult offspring: a cross‐sectional study in a resource‐limited setting in northern Peru
Source: Trop Med Int Health. 2018 Apr 1;23(5):533–40. doi: 10.1111/tmi.13052 (PMC5932220; doi:10.1111/tmi.13052)
Supplement: Supplementary file 2 — Appendix S1. Fitted Regression Model. [file TMI-23-533-s002.docx]

## Supplementary Material 1: Fitted Regression Model.

The fitted model for offspring BMI (it was similar for SBP and DPB) was:

$${}{}\left( \right){}\left( \right){}\left( \right){}\left( \right){}\left( \right){}\left( \right){}\left( \right){}\left( \right){}{BMI}_{ij}=\beta_{0}+ \beta_{1}{FATHER}_{j}+\beta_{2}{MOTHER}_{j}+ \beta_{3}\left( {BMI FATHER}_{j} \right)+\beta_{4}\left( {BMI MOTHER}_{j} \right)+\beta_{5}{SEX}_{ij}+ \beta_{6}\left( {BMI FATHER}_{j}\times{SEX}_{ij} \right)+ \beta_{7}\left( {BMI MOTHER}_{j}\times{SEX}_{ij} \right) {}{}{}{}+{COVARIATES}_{ij}+ \alpha_{j}+\epsilon_{ij}$$

where $\alpha_{j}\sim N(0,\sigma_{\alpha}^{2})$ is the random error associated with family $j$ and $\epsilon_{ij}\sim N(0,\sigma^{2})$ is the random error associated with the observation ${BMI}_{ij}$ where *i =1…442* and *j = 1…308*. Outcomes and exposures were defined as

- ${BMI}_{ij}$ is the BMI value of the offspring $i$ from the family $j$
- ${FATHER}_{j}$and ${MOTHER}_{j}$are an indicator variable of whether or not the father (mother) lived with the family.
- ${BMI MOTHER}_{j}$ (${BMI FATHER}_{j}$) is the value of the BMI of the mother (father) in the family $j$and when there was no a mother (father) in the family.


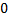


- ${SEX}_{ij}$ is the sex of the offspring $i$ from the family $j$ (0 female and 1 male)

Given that a family has a father present, and with the rest of covariates fixed the mean BMI levels of the offspring is given by

$$\beta_{0}+ \beta_{1}+\beta_{3}\left( {BMI FATHER}_{j} \right)+\beta_{5}\left( {BMI FATHER}_{j}\times{SEX}_{ij} \right)$$

Then the effect of BMI of the father on female offspring is capture by $\beta_{3}$ and the effect of BMI of the father on a male offspring is capture by $\beta_{3}+ \beta_{5}$. Ninety five percent confidence intervals for the combination of coefficients were estimated with the post-estimators linear combinations (*lincom* command in STATA).

This table show the way the key parameters are computed from the model

|  |  | Expected difference in BMI |
| --- | --- | --- |
| Father - Female Offspring | ${}$ | $\beta_{3}$ |
| Father - Male Offspring | ${}{}$ | $\beta_{3}+\beta_{5}$ |
| Mother - Female Offspring | ${}$ | $\beta_{4}$ |
| Mother - Male Offspring | ${}{}$ | $\beta_{4}+\beta_{6}$ |

$${}{}{}{}{}{}{}{}{}$$
